# A Novel Automated Approach for Improving Standardization of the Marble Burying Test Enables Quantification of Burying Bouts and Activity Characteristics

**DOI:** 10.1523/ENEURO.0446-21.2022

**Published:** 2022-03-31

**Authors:** Lucas Wahl, A. Mattijs Punt, Tara Arbab, Ingo Willuhn, Ype Elgersma, Aleksandra Badura

**Affiliations:** 1 Department of Neuroscience, Erasmus University Medical Center Rotterdam, 3015 GD Rotterdam, The Netherlands; 2 Department of Clinical Genetics, Erasmus University Medical Center Rotterdam, 3015 GD Rotterdam, The Netherlands; 3 Netherlands Institute for Neuroscience, Royal Netherlands Academy of Arts and Sciences, Amsterdam, 1105 BA Amsterdam, The Netherlands; 4 Department of Psychiatry, Amsterdam University Medical Centers, University of Amsterdam, Amsterdam, 1105 AZ Amsterdam, The Netherlands

**Keywords:** activity characteristics, anxiolytics, automated classification, burying characteristics, marble burying test, open-source tools

## Abstract

The marble burying test is a commonly used paradigm to describe phenotypes in mouse models of neurodevelopmental and psychiatric disorders. The current methodological approach relies predominantly on reporting the number of buried marbles at the end of the test. By measuring the proxy of the behavior (buried marbles), many important characteristics regarding the temporal aspect of this assay are lost. Here, we introduce a novel, automated method to quantify mouse behavior during the marble burying test with the focus on the burying bouts and movement dynamics. Using open-source software packages, we trained a supervised machine learning algorithm (the “classifier”) to distinguish burying behavior in freely moving mice. In order to confirm the classifier’s accuracy and characterize burying events in high detail, we performed the marble burying test in three mouse models: *Ube3a^m-/p+^* [Angelman syndrome (AS) model], *Shank2*^−/−^ (autism model), and *Sapap3*^−/−^ [obsessive-compulsive disorder (OCD) model] mice. The classifier scored burying behavior accurately and consistent with the previously reported phenotype of the *Ube3a^m-/p+^* mice, which showed decreased levels of burying compared with controls. *Shank2*^−/−^ mice showed a similar pattern of decreased burying behavior, which was not found in *Sapap3*^−/−^ mice. Tracking mouse behavior throughout the test revealed hypoactivity in *Ube3a^m-/p+^* and hyperactivity in the *Shank2*^−/−^ mice, indicating that mouse activity is unrelated to burying behavior. Reducing activity with midazolam in *Shank2*^−/−^ mice did not alter the burying behavior. Together, we demonstrate that our classifier is an accurate method for the analysis of the marble burying test, providing more information than currently used methods.

## Significance Statement

The marble burying test is widely used in phenotyping neurodevelopmental and neuropsychiatric disorder mouse models. Currently, its analysis consists largely of manually scoring the number of buried marbles on the completion of the assay. This approach is not standardized across laboratories, and leaves out important variables such as movement characteristics and information about the burying bouts. We introduce a method that reliably tracks mouse behavior throughout the experiment, classifies the duration and number of the burying bouts, and is generalizable across laboratories. Using machine learning for measuring the actual burying behavior standardizes this method, and provides rich information about the burying characteristics and overall behavior.

## Introduction

The marble burying test (Pinel and Treit, 1978) is a commonly used paradigm, aimed at studying repetitive behavior as well as an anxiety-like phenotype (Broekkamp et al., 1986; Thomas et al., 2009; Angoa-Pérez et al., 2013). It has more recently been used to study models of neuropsychiatric and neurodevelopmental disorders, with over 87% of studies being done in mice (Çalişkan et al., 2017). The behavioral meaning of marble burying is however highly debated throughout literature. Studies have found that burying behavior can be selectively inhibited by some anxiolytics and antidepressants (Ichimaru et al., 1995; Nicolas et al., 2006; Wise et al., 2012) in a dose-dependent manner. However, results show no correlation with “anxiety-related” responses in the open-field or light–dark tests (such as elevated plus maze), nor are they correlated with overall exploratory activity (Thomas et al., 2009). Marble burying and digging-event frequency were found to correlate only on the first 2 out of 5 d of repeated testing, showing a dissociation between burying and digging behavior (Thomas et al., 2009; Taylor et al., 2017). Same studies show that mice tend to decrease the number of buried marbles when tested multiple times within 1 d.

The most commonly used scoring method focuses only on the number of buried marbles. In this classical approach, mice are removed from the apparatus at the end of testing and an experimenter assesses visually how many marbles are covered more than a chosen threshold. This threshold varies throughout literature but is commonly set at a marble being either 50% or two-thirds covered by bedding to be considered buried (Thomas et al., 2009; Angoa-Pérez et al., 2013; Kalariya et al., 2015; Sonzogni et al., 2019). The main benefit of this method is high throughput because of the short analysis time needed. It has also been shown to be highly consistent within a given mouse model (Sonzogni et al., 2018). This visual assessment method is used in the vast majority of published studies (de Brouwer et al., 2019). However, several research groups added additional analysis in the efforts to better describe the marble burying results. The most common adaptations are: (1) *post hoc* manual assessment of photographs taken before and after the test (Homma and Yamada, 2009); (2) analysis of the interobserver reliability (Kinsey et al., 2011); (3) automated tracking of mouse mobility during the test (Nicolas et al., 2006; Serra et al., 2021). Although these methods indeed provide additional information about mouse behavior, they are not widely adopted and they do not quantify the spatio-temporal characteristics of the burying bouts. A few studies, which report burying and digging events, label them by manually annotating the video frames (Wright-Williams et al., 2013; Smith et al., 2014; Serra et al., 2021), which is an accurate but highly time-consuming method.

Automated classification of behaviors based on machine learning algorithms provides a way to study animal behaviors over time in high detail (Kabra et al., 2013; Pereira et al., 2019; Wiltschko et al., 2020). In the last few years, this technique has been used to identify social and locomotor behaviors in mice (van den Boom et al., 2017), and other species (Aso et al., 2014; Blut et al., 2017). Although lower throughput than visual inspection, the collected videos can be analyzed in a batch mode, significantly speeding up the analysis process, and once trained, the classifiers can be used across many experiments.

Here, we used supervised machine learning to train a classifier to provide a method for repeatable inter-experimenter and intra-experimenter scoring of burying behavior that gives additional information regarding spatial and temporal burying characteristics. To test the accuracy of the classifier, we evaluated whether it was adept at detecting the established burying phenotype in a mouse model of Angelman syndrome (AS; *Ube3a^m-/p+^*; Huang et al., 2013; Sonzogni et al., 2018; Wang et al., 2018). Using an automated video tracking software in combination with our classifier we were able to detect the clear hypoactivity phenotype of the *Ube3a^m-/p+^* mice and determine the spatial characteristics of the burying events.

Furthermore, we used our classifier to describe the characteristics of burying behavior in the *Shank2*^−/−^ model of autism spectrum disorder (ASD; Schmeisser et al., 2012; Won et al., 2012) and the *Sapap3*^−/−^ model of obsessive-compulsive disorder (OCD; Welch et al., 2007), two additional mouse models known for repetitive and compulsive-like/anxious behaviors, respectively. We observed that burying behavior was not increased for *Shank2*^−/−^ and *Sapap3*^−/−^ mice. Additionally, burying behavior was found to be independent of locomotor activity patterns in those models since application of an anxiolytic drug, midazolam, decreased the hyperactivity phenotype in *Shank2*^−/−^ mice but did not significantly alter the burying behavior. Our novel method provides rich information about the burying characteristics and overall behavior and can be adapted to many experiments across different laboratory settings.

## Materials and Methods

### Experimental procedures

All experimental animal procedures were approved *a priori* by an independent animal ethical committee (DEC-Consult, Soest, The Netherlands), as required by Dutch law and conform to the relevant institutional regulations of the Erasmus Medical Center, the Netherlands Institute for Neuroscience KNAW, and Dutch legislation on animal experimentation (CCD approval: AVD1010020197846, AVD101002016791, and AVD801002015126).

### Animals

We used male and female mice of the following strains: (1) *Ube3a ^m-/p+^* (*Ube3a^tm2Yelg^*; Wang et al., 2018) mice (27.13 ± 0.78 g, seven males and five females) and their wild-type (WT) littermates (24.07 ± 1.03 g, six males and six females); (2) *Shank2*^−/−^ mice (20.1 ± 0.97 g, five males and five females for standard marble burying tests and eight males for pharmacological testing) and their WT littermates (22.1 ± 0.69 g, five males and five females; Schmeisser et al., 2012; Won et al., 2012); (3) *Sapap3*^−/−^ mice (eight males and four females) and their WT littermates (10 males and two females, Welch et al., 2007). Strain 1 was generated in the F1 hybrid 129S2-C57BL/6J background. Strains 2 and 3 were bred on a C57BL/6J background.

Strains 1–2 were between 8 and 12 weeks of age and were housed and tested in the Erasmus Medical Center Rotterdam, The Netherlands. Animals had *ad* *libitum* access to water and food (standard laboratory chow) and were kept on a regular 12/12 h light/dark cycle. *Shank2*^−/−^ mice and their WT littermates were group-housed (three mice per cage, mixed genotypes in the same cage). *Ube3a^m-/p+^* mice and their WT littermates were group-housed (three mice per cage, mixed genotypes in the same cage). All mice from strains 1–2 were kept on wood chip bedding (Lignocel Hygienic Animal Bedding, JRS), with a density of 204 g/l, which was also used for the experiments.

*Sapap3*^−/−^ mice as well as their WT littermates (strain 3) were between 8 and 18 weeks of age and were housed and tested at the Netherlands Institute for Neuroscience, Amsterdam, The Netherlands. Animals had *ad libitum* access to water and food (standard laboratory chow) and were group housed on a regular 12/12 h light/dark cycle. The *Sapap3*^−/−^ mice and their littermates were kept on corn-cob bedding (Bio Services EuroCob Corn), with a density of 566 g/l, that was also used for the experiments.

### Behavioral testing

Animals were habituated to the testing room for at least 1 h before experiments. To isolate external factors, all experiments were done inside a 130 × 80 × 80 cm wooden box with a door. The 6 mm high-pressure laminate walls were lined with acoustic foam to reduce external noise penetration. Testing was done in a 26.6 × 42.5 × 18.5 cm cage (Eurostand 1291H-type III H) which rested on an elevated 10-mm frosted Perspex shelf. The apparatus was evenly lit from top and bottom using white LED strips and recorded with an overhead camera (Basler acA1300-600gm) with a 4.4–11 mm/F1.6ens (KOWA) at 25 frames per second. Testing cages were filled with wood chip bedding (Lignocel Hygienic Animal Bedding, JRS) for strains 1–2, and corn-cob bedding (Bio Services EuroCob Corn) for strain 3, to the height of ∼4 cm. Next, 20 blue glass marbles were spaced out evenly in four rows on the bedding. The recordings were started immediately after the animals were placed in the cage, using a custom Bonsai script (Lopes et al., 2015). The animals were left to explore the apparatus for 30 min, after which the mice were removed and a top-down image of the marbles was taken. The bedding was discarded and the cages were cleaned with 70% ethanol between each experiment.

### Pharmacological testing

Animals were acclimated to the testing room for at least 1 h before experiments. Midazolam (1 mg/kg) was dissolved in physiological saline (0.9%). Injections were given through the intraperitoneal route of administration in a volume of 5 μl/g of body mass. On day 1 of pharmacological testing, four mice received a saline injection whereas the other four mice received a midazolam injection. Animals received intraperitoneal injections 30 min before the test. Following the marble burying test, animals were given 1 d of rest. On day 3, mice that previously received saline injections were injected with midazolam, and mice that previously received midazolam injections received a saline injection, allowing for within-animal saline-dug comparison.

### Manual scoring of burying behavior

Manual annotation of four 10-min videos was done by four observers using the open-source software BORIS (Friard and Gamba, 2016), which allowed for frame-by-frame annotation.

### Classifier training

A classifier to study burying behavior was created in the open source, MATLAB (MathWorks, R2018a) based Janelia Automatic Animal Behavior Annotator (JAABA; v0.6) environment (Kabra et al., 2013). Videos were prepared by cropping raw images to the area of the apparatus using FFMPEG (https://www.ffmpeg.org/). To prepare the data for classifier training, the videos were then tracked using open-source software MATLAB based Mouse Tracker (motr; Ohayon et al., 2013). All videos were processed and tracked in a batch-mode, significantly decreasing the processing time. The output data from motr were converted to the required format for JAABA by using the function “PrepareJAABA.” Version 0.6.0 of JAABA was obtained from SourceForge (http://jaaba.sourceforge.net/). The classifier was trained on 13,203 frames in three videos of *Shank2*^−/−^ mice and two videos of their WT littermates. Not all frames were annotated to get a relatively equal distribution of burying and non-burying frames. A minimum bout length of one second was applied for the analysis of burying characteristics. The trained classifier is available at https://doi.gin.g-node.org/10.12751/g-node.syheka/. However, to obtain maximum accuracy, we recommend training a custom classifier on newly acquired videos as the experimental conditions such as light, cage dimensions, mouse color and bedding can differ across laboratories. The classifier can then be used for all newly acquired videos.

To obtain information regarding movement characteristics, all videos were tracked using open-source software Optimouse (Ben-Shaul, 2017). All videos were tracked in a batch-mode, which significantly decreased the processing time. The (x,y) position data from Optimouse were combined with the frame by frame output of JAABA to create heatmaps of burying topography using a custom-written MATLAB script.

### Image analysis

A custom script to analyze buried marble surface area was created in ImageJ (https://imagej.nih.gov/ij/). The marbles are separated from the background with the use of color thresholding, after which the masked-out surface area can be measured per marble.

### Code accessibility

The code/software described in the paper is freely available online at https://github.com/BaduraLab/Marble-Burying. The ImageJ code is available as Extended Data 1. The classifier is deposited at https://doi.gin.g-node.org/10.12751/g-node.syheka/.

10.1523/ENEURO.0446-21.2022.ed1 Extended Data 1 Marble surface area color macro. Download Extended Data 1, TXT file.

### Data processing and statistics

All data were processed using Microsoft Excel and custom MATLAB scripts, on a Windows 10 64-bit computer. Statistical group comparisons were done using GraphPad Prism 8 software. The assumption of normality was tested using the D’Agostino–Pearson test. For pharmacological testing the Shapiro–Wilk test was used to determine normality. For nonpaired data, if the data passed the assumption of normality, a one-tailed or two-tailed *t* test was used to compare groups. If the assumption of normality was violated, a one-tailed or two-tailed Mann–Whitney test was used. For paired data, if the data passed the assumption of normality, a paired *t* test was used. If the assumption of normality was violated, a Wilcoxon test was used.

## Results

### Classification performance

**Figure 1.**
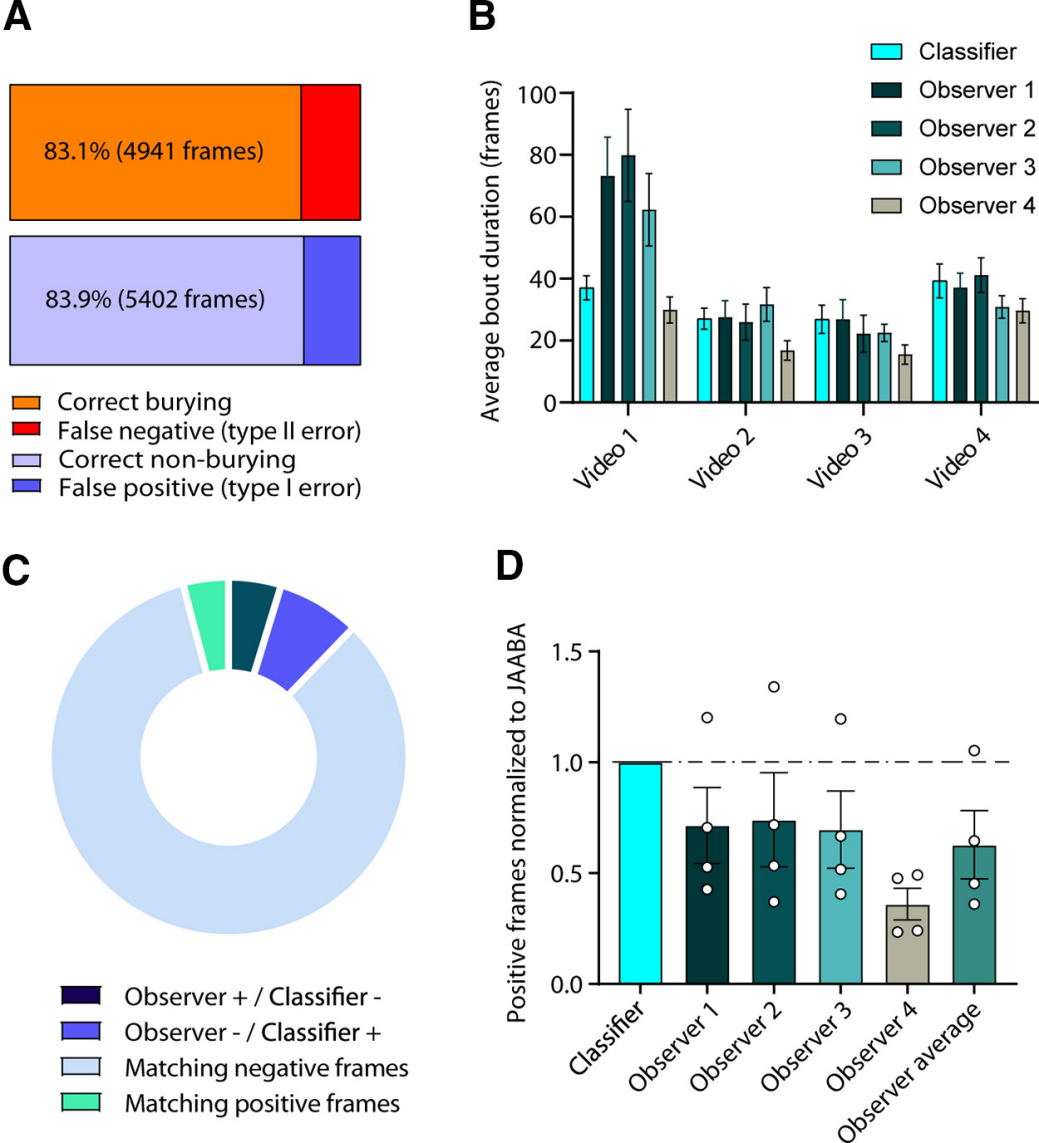
Classifier validation. ***A***, Cross-validation results of 13,203 manually-annotated frames. The orange bar represents correct annotation of frames containing burying, while red represents frames incorrectly scored as burying by the classifier. Light-blue bar represents correct annotation of non-burying frames, whereas dark-blue shows incorrectly annotated non-burying frames. ***B***, Average duration of bouts in four videos of 10 min in duration, manually scored by four observers. ***C***, Pie chart depicts (1) frames that observers scored as burying, whereas the classifier scored non-burying (Observer + / Classifier −); (2) frames that observers scored as non-burying, whereas the classifier scored burying (Observer − / Classifier +); and (3/4) non-burying and burying frames where observers were in consensus with the classifier (Matching positive/negative frames). ***D***, Frames scored positive for burying by the observers normalized to the results of the classifier. Each dot represents a single video annotated by an observer.

We collected five videos of marble burying (30 min/25 fps each) from three male *Shank2*^−/−^ and two female *Shank2*^−/−^ mice of 14–18 weeks old in our custom-built marble burying setup (for details, see Materials and Methods). We preprocessed the videos by cropping them to the size of the marble burying arena using FFMPEG (https://www.ffmpeg.org/) and subsequently tracked the mice using open source Mouse Tracker (motr; Ohayon et al., 2013). Next, we transferred the videos to the JAABA environment (Kabra et al., 2013) and trained the classifier to discriminate the burying events. Cross-validation of frames labeled by experimenters during training of the classifier showed that the classifier achieved a classification accuracy of 83.1% for correctly annotating burying frames and 83.9% for non-burying frames (Fig. 1*A*). Manual annotation of four 10-min videos by four independent observers blinded to genotype showed high variability in the reported average bout duration between observers (Fig. 1*B*). There was a significant overlap in the annotation made by the observers and the classifier (Fig. 1*C*). Overall, the observers annotated less frames as burying-positive than the classifier (Fig. 1*D*).

### Classification validation in the Angelman mouse model

In order to validate the results from the classifier, we chose a mouse model with a well-documented phenotype in the marble burying test. *Ube3a* mutant mice (*Ube3a^m-/p+^*) are a model for AS, which have consistently shown impaired marble burying behavior (Sonzogni et al., 2018; Rotaru et al., 2020).

**Figure 2.**
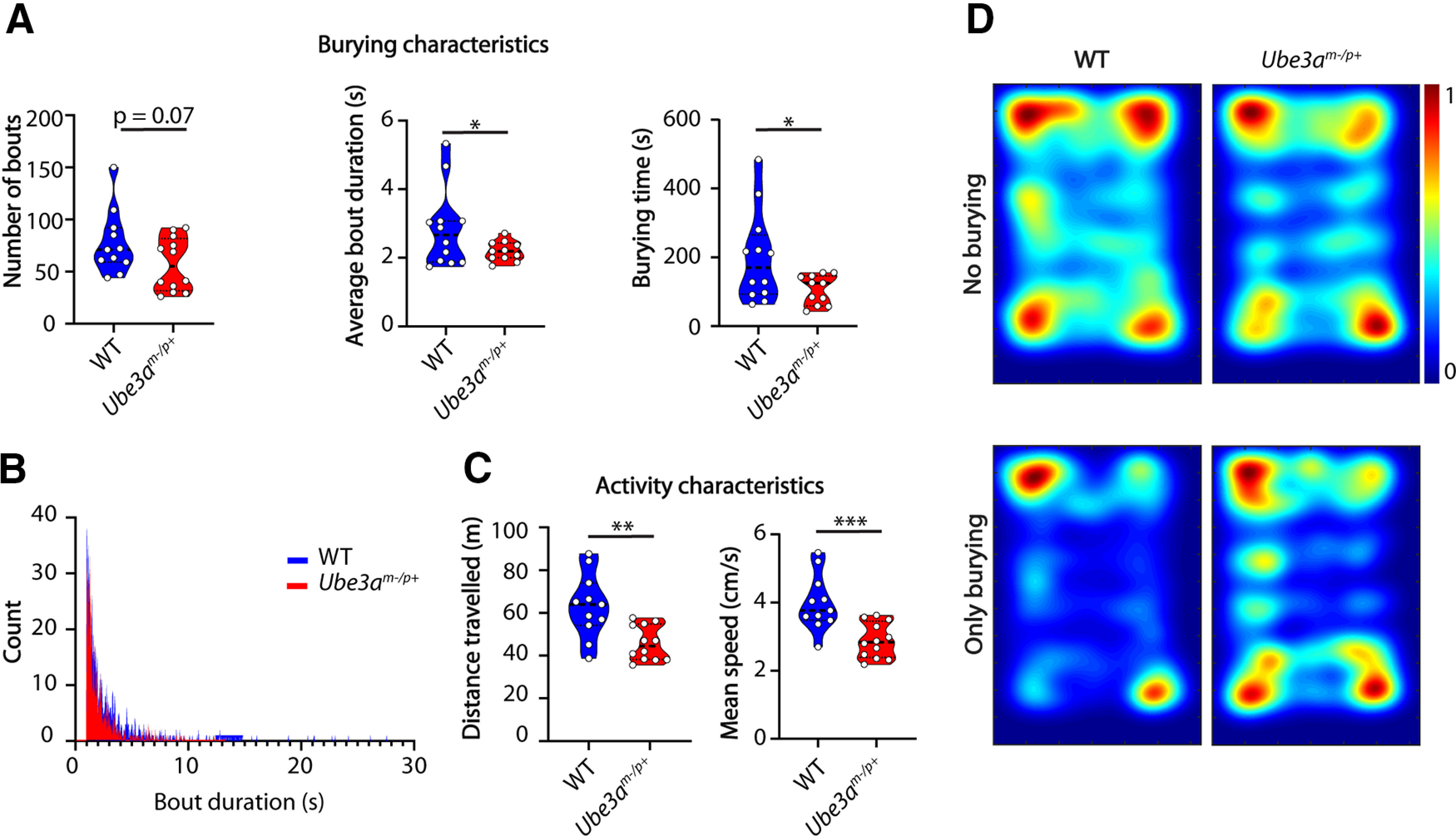
*Ube3a* mutants show a burying phenotype consistent with literature. ***A***, Number of bouts, average bout duration, and total burying time for WT and *Ube3a^m-/p+^* mice during the marble burying test. Data presented as median with interquartile range (number of bouts, one-tailed Mann–Whitney test; average bout duration and total burying time, two-tailed, unpaired *t* test). ***B***, Histogram showing distribution of bout lengths and their frequency. ***C***, Distance traveled and mean speed over the duration of the test. Data presented as median with interquartile range (one-tailed, unpaired *t* test). ***D***, Heatmaps showing all frames where mice do not bury (top) and frames where mice show burying behavior (bottom); *p ≤ 0.05, **p ≤ 0.01, ***p ≤ 0.001, *n* = 12 mice per genotype except for the WT group where *n* = 11 for ***A***, ***B***, ***D*** because of erroneous motr tracking coming from an artifact of the experimenter’s hand in the field of view.

All videos were collected and processed as described above and in Materials and Methods. Using our classifier, we indeed found that *Ube3a^m-/p+^* mice (*n* = 12) spent less time burying than their WT littermates (*n* = 11; *p* = 0.0175), which was caused by a trend in the decreased number of bouts (*p* = 0.0735) as well as shorter average bout duration (*p* = 0.0405; Fig. 2*A*,*B*). Next, we used open-source software Optimouse (Ben-Shaul, 2017) to analyze movement characteristics independent of the burying behavior. *Ube3a^m-/p+^* mice traveled significantly less distance (*p* = 0.0011) during the marble burying test and were significantly slower (*n* = 12 both genotypes; *p* = 0.0004; Fig. 2*C*), showing decreased locomotor activity consistent with previous findings (Sonzogni et al., 2018). By combining the output of the tracking software with the output of the classifier we created heatmaps illustrating spatial information regarding burying events. Both *Ube3a^m-/p+^* mice as well as their WT littermates had a strong preference for moving in the corners of the apparatus (Fig. 2*D*, top). However, while the WT mice predominantly buried in the corners, the burying behavior of *Ube3a^m-/p+^* mice was less spatially specific (Fig. 2*D*, bottom).

### Quantification of complex parameters reveals unique features of marble burying behavior

Burying behavior in the marble burying test is often ascribed to anxiety-like behaviors and/or repetitive behaviors, but conclusive evidence for either type of behavior is lacking. Because our methodological approach enables measuring several behavioral parameters in one assay, we performed the marble burying experiments with two additional mouse models to study this further.

**Figure 3.**
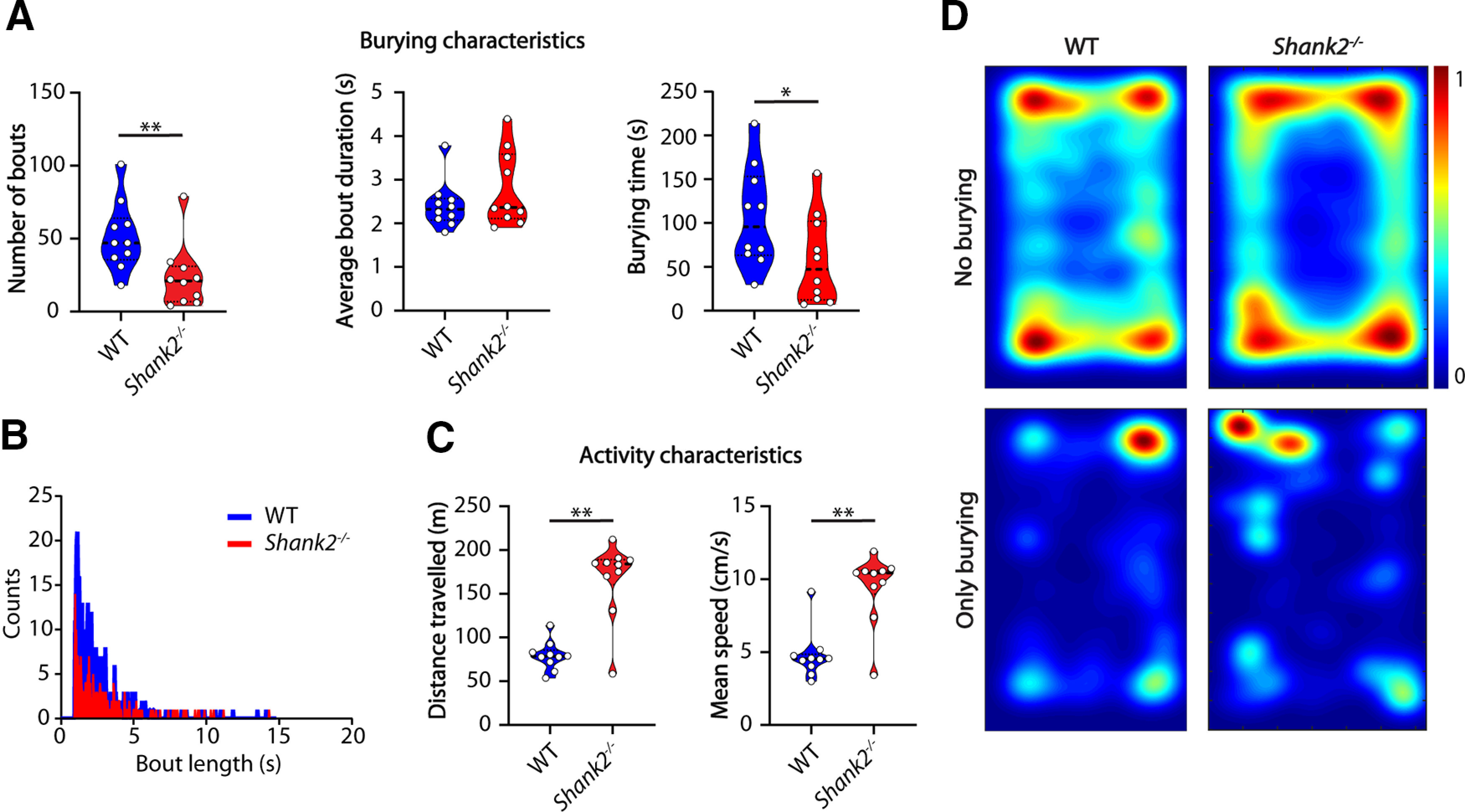
*Shank2* mutants show decreased number of bouts but similar bout duration compared with WTs. ***A***, Number of bouts, average bout duration, and total burying time shown for WT and *Shank2* mutant mice during the marble burying test. Data presented as median with interquartile range (number of bouts and average bout duration, one-tailed Mann–Whitney test; burying time, one-tailed, unpaired *t* test). ***B***, Histogram showing distribution of bout lengths and their frequency. ***C***, Distance traveled and mean speed over the duration of the test. Data presented as median with interquartile range (one-tailed Mann–Whitney test). ***D***, Heatmaps showing all frames where mice do not bury (top) and frames where mice do bury (bottom); *p ≤ 0.05, **p ≤ 0.01; *n* = 10 mice per genotype.

We first tested mice with a mutation in the *Shank2* gene, an established mouse model for ASD (Peter et al., 2016; Eltokhi et al., 2018; Kim et al., 2018), that consistently display increased repetitive behavior in the grooming assay and hyperactivity (Schmeisser et al., 2012; Peter et al., 2016). In the marble burying test however, *Shank2*^−/−^ mice (*n* = 10 mice per genotype) showed a significant decrease in the number of burying bouts (*p* = 0.0032) and total burying time (*p* = 0.0309; Fig. 3*A*). There was no difference in average bout length (*p* = 0.2894), reflected in no visible difference in shape of the distribution of bout durations (Fig. 3*B*). Therefore, we can conclude that the decrease in the number of burying bouts was evenly distributed across shorter and longer bouts. The hyperactivity phenotype was clearly present in the marble burying test as *Shank2* mutants traveled larger distances (*p* = 0.0005) at a higher speed (*p* = 0.0008; Fig. 3*C*). *Shank2*^−/−^ mice hyperactivity was evident from the heatmaps, with minor differences in spatial distribution of the burying events when compared with WT littermates (Fig. 3*D*).

**Figure 4.**
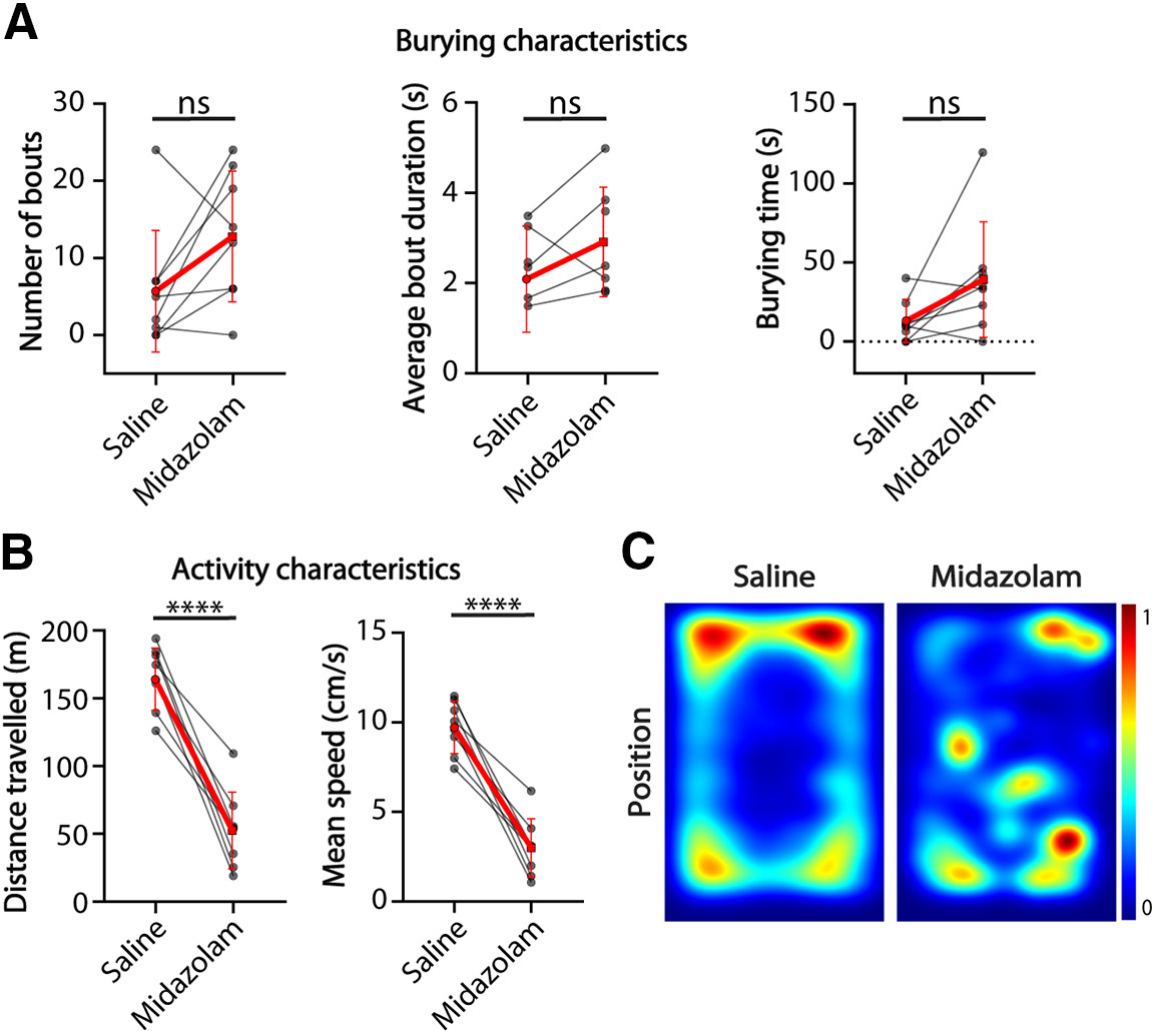
*Shank2* mutants show decreased locomotion on midazolam treatment but similar burying characteristics compared with saline controls. ***A***, Number of bouts, average bout duration, and total burying time shown for *Shank2* mutant mice during the marble burying test. Red lines indicate group mean with SD (number of bouts, Wilcoxon test; average bout duration and burying time, two-tailed paired *t* test). ***B***, Distance traveled and mean speed over the duration of the test. Red lines indicate group mean with SD (two-tailed paired *t* test). ***C***, Heatmaps showing the position of the mice during the test after saline injection (left) and midazolam injection (right); ****p ≤ 0.0001; ns = not significant; *n* = 8 mice.

To further test how hyperactivity might influence the burying behavior in *Shank2*^−/−^ mice and whether decreased burying behavior can be rescued by decreasing locomotor activity, we assessed within-animal performance change in the marble burying test as a result of midazolam injection in eight *Shank2*^−/−^ mice. Midazolam has previously been shown to reduce locomotor activity and marble burying in mice (Wise et al., 2012). The number of burying bouts (*p* = 0.0938), average bout duration (*p* = 0.3026) and total burying time (*p* = 0.1533) were not affected significantly after midazolam injection (Fig. 4*A*). *Shank2*^−/−^ mice traveled significantly less distance (*p* < 0.0001) after midazolam administration and were significantly slower (*p* < 0.0001; Fig. 2*C*), showing decreased locomotor activity. Heatmaps indicate a reduced preference for the corners of the arena, consistent with the anxiolytic effects of midazolam (Wise et al., 2012).

**Figure 5.**
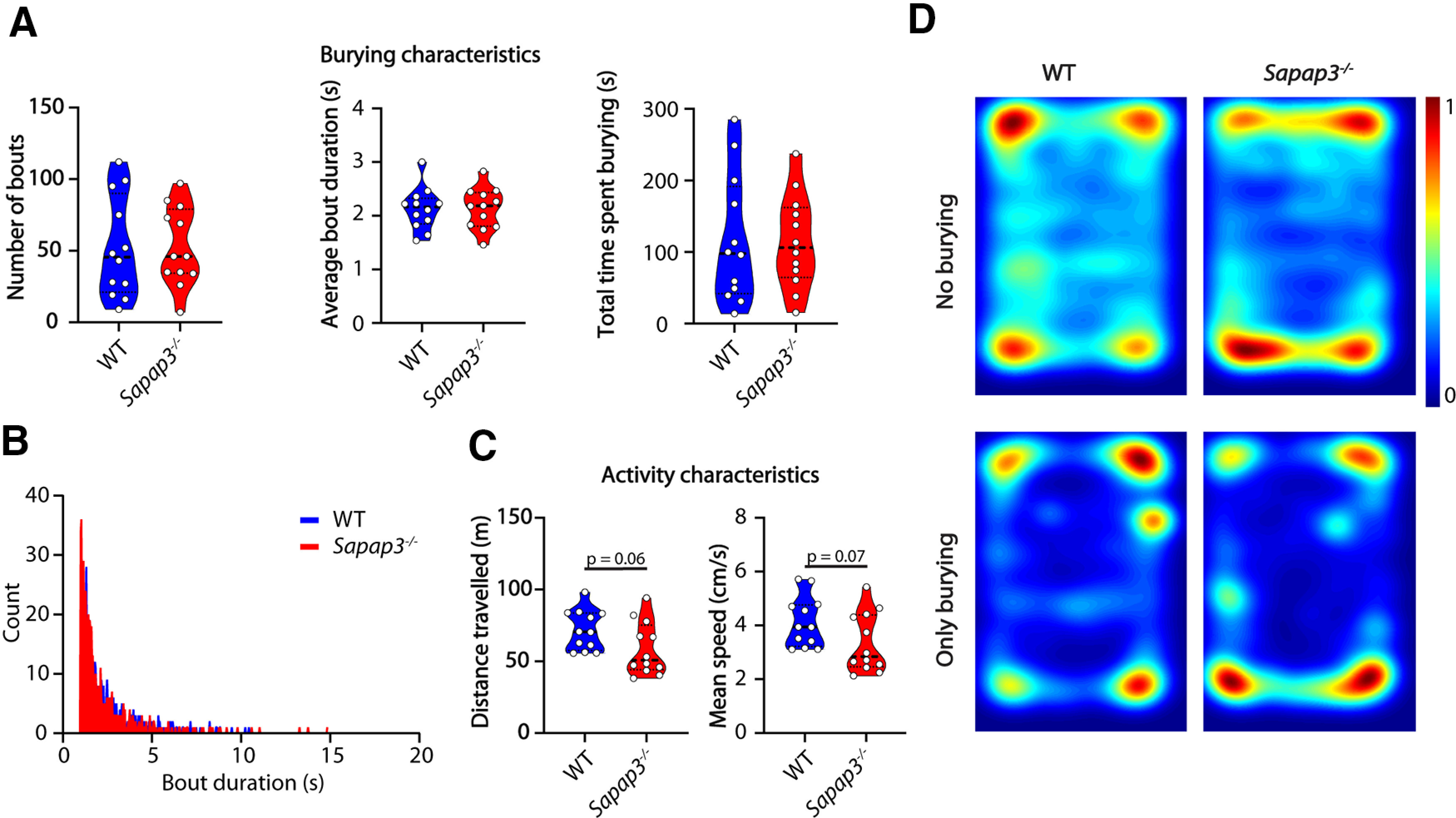
*Sapap3*^−/−^ mice do not show a burying phenotype but tend to travel less distance and do so at decreased speeds. ***A***, Number of bouts, average bout duration, and total burying time shown for WT mice and *Sapap3*^−/−^ mice during the marble burying test. Data presented as median with interquartile range (two-tailed, unpaired *t* test). ***B***, Histogram showing distribution of bout lengths and their frequency. ***C***, Distance traveled and mean speed over the duration of the test. Data presented as median with interquartile range (two-tailed, unpaired *t* test). ***D***, Heatmaps showing all frames where mice do not bury (top) and frames where mice do bury (bottom); *n* = 12 mice per genotype.

We next examined the performance of *Sapap3*^−/−^ mice in the marble burying test. *Sapap3*^−/−^ mice present with a phenotype that matches considerably with OCD patients, including compulsive-like grooming, decreased cognitive flexibility, altered habit formation, and increased anxiety-like behavior (Welch et al., 2007; van den Boom et al., 2019; Ehmer et al., 2020b). Similar to OCD patients, compulsive grooming present in *Sapap3*^−/−^ mice can be rescued by administration of selective serotonin reuptake inhibitors (SSRIs) or deep-brain stimulation (Welch et al., 2007; Pinhal et al., 2018). However, in the marble burying assay the *Sapap3*^−/−^ mice did not show altered burying characteristics: there was no difference between the mutant mice and their WT littermates (*n* = 10 mice per genotype) in number of bouts (*p* = 0.9441), bout duration (*p* = 0.9856), total time spent burying (*p* = 0.9362) or bout-duration distribution (Fig. 5*A*,*B*). *Sapap3*^−/−^ mice showed a tendency to travel less and at lower speeds (Fig. 5*C*). Consistent with the anxiety phenotype (Welch et al., 2007) they spent most time in the corners (Fig. 5*D*, top), but the spatial distribution of the burying events was not affected.

**Figure 6.**
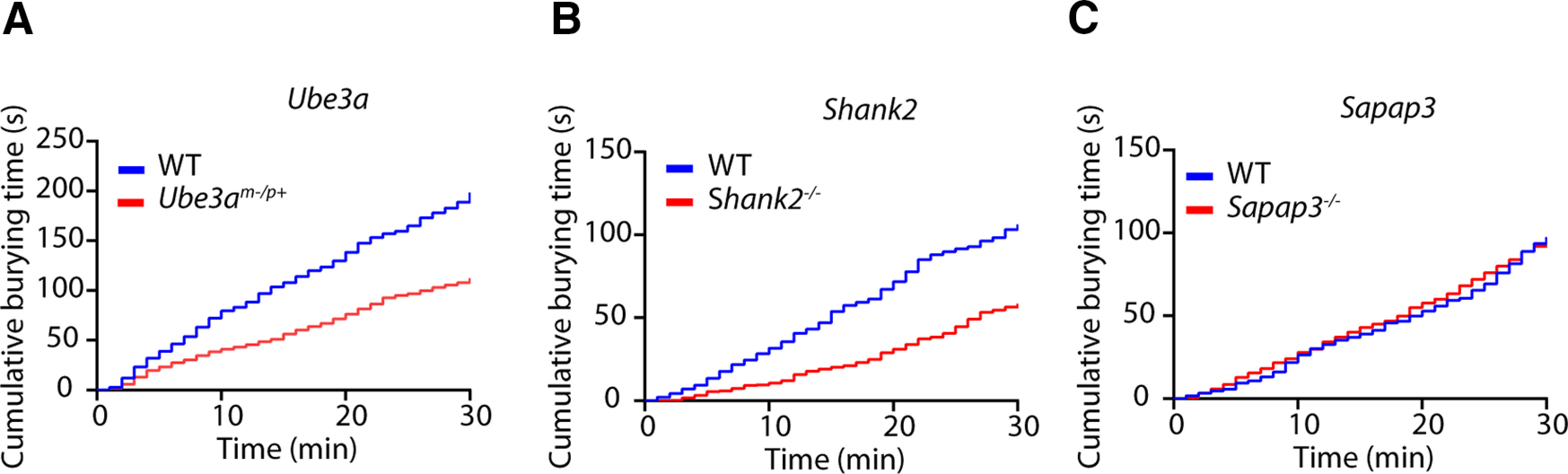
Cumulative burying over time. Time-binned plot with cumulative burying over time. Each bin represents a 1-min time period. Groups shown are *Ube3a^m-/p+^* (***A***), *Shank2*^−/−^ (***B***), and *Sapap3*^−/−^ (***C***), and their respective control littermates; *n* = 12 mice per genotype for ***A***, ***C*,** *n* = 10 mice per genotype for ***B***.

Our methodological approach allowed us to quantify the duration and characteristics of the burying events as well as their distribution in time (Fig. 6). *Ube3a^m-/p+^* and *Shank2*^−/−^ mice showed a consistent decrease in burying behavior over the entire session (Fig. 6*A*,*B*) compared with control mice. No difference was found for *Sapap3*^−/−^ mice (Fig. 6*C*).

### Comparison of analysis methods

**Figure 7.**
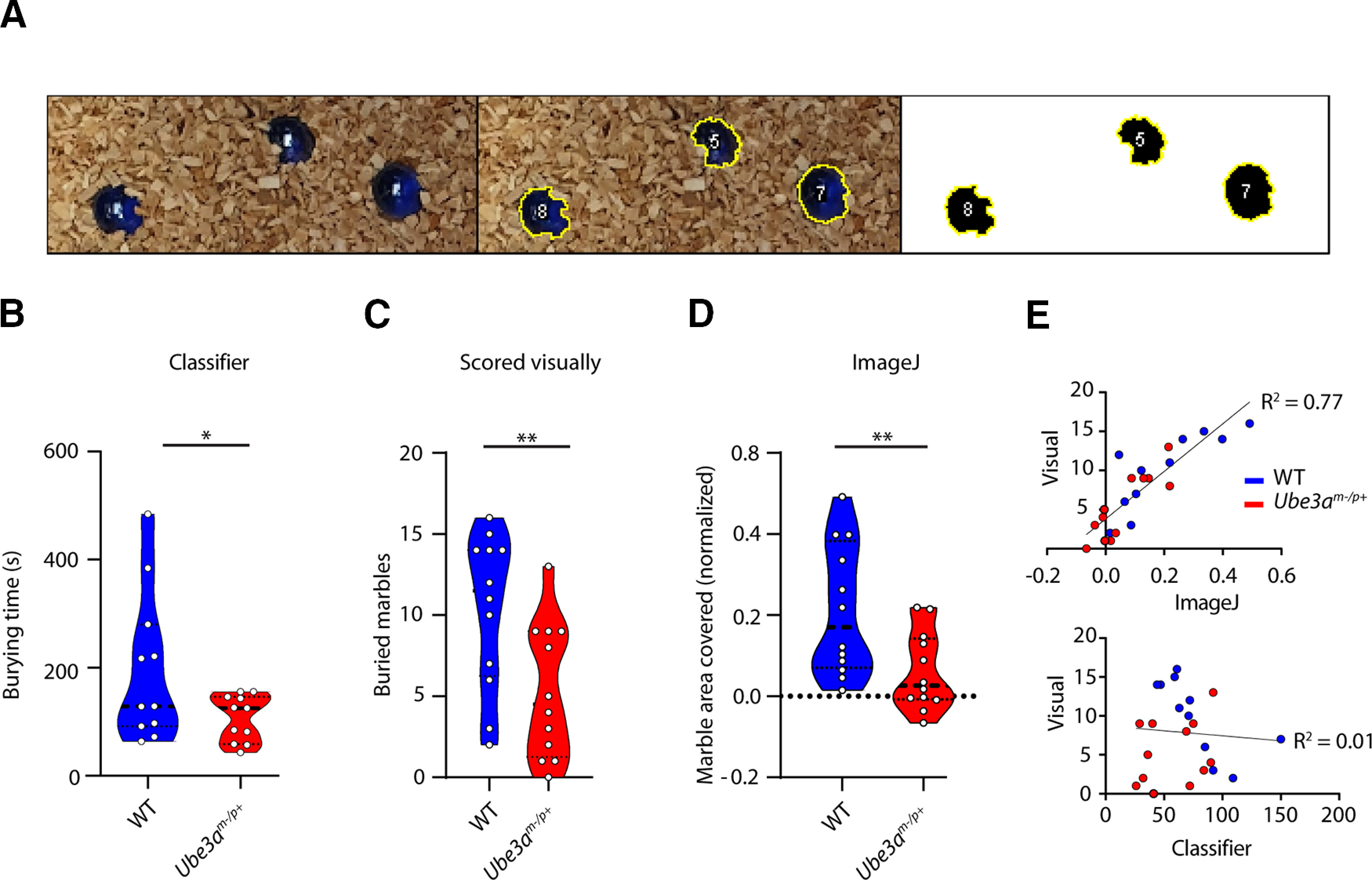
Analysis method comparison. ***A***, Example of thresholded marbles analyzed with the ImageJ script. Analysis was done by taking a photograph of the buried marbles at the end of each test (left), color thresholding the marbles (middle), and measuring the masked surface area (right). ***B***, *Ube3a^m-/p+^* results as analyzed with the trained classifier. Data presented as median with interquartile range (one-tailed, unpaired *t* test). ***C***, Same mice as in ***A***, but analyzed using visual scoring at the end of each test by the experimenters. Data presented as median with interquartile range (one-tailed, unpaired *t* test). ***D***, Same mice as in ***A***, ***B***, but analyzed with the ImageJ script. Shown is the marble area that is left uncovered. Data presented as median with interquartile range (one-tailed, unpaired *t* test). ***E***, Scatterplots showing correlation between ImageJ and visual scoring (top) and correlation between the classifier and visual scoring (bottom); *p ≤ 0.05, **p ≤ 0.01; ***A***, *n* = 11 for WT and *n* = 12 for *Ube3a^m-/p+^*; ***B***, ***C***, *n* = 12 per genotype.

In order to provide a standardized alternative, fast way of analyzing the result of the marble burying test other than visual scoring, we developed a script in ImageJ to measure buried surface area per marble, based on color thresholding of the marbles and measuring the masked surface area (Fig. 7*A*). We found that the results of the *Ube3a^m-/p+^* mice and their WT littermates were comparable between the classifier (*p* = 0.0175; Fig. 7*B*), experienced visual experimenter scoring (*p* = 0.0061; Fig. 7*C*), and image analysis (*p* = 0.0056; Fig. 7*D*). Visual scoring results per animal showed a strong correlation with the analysis of postburying images using ImageJ (Fig. 7*E*, top). However, burying behavior scored by the classifier showed no direct correlation with the number of buried marbles (Fig. 7*E*, bottom). Although no information is gained over burying characteristics over time, measuring buried marble surface area with ImageJ provides a way to analyze the marble burying result in a way that is similarly fast as visual scoring, yet with replicable results that are not based on experimenter performance or experience.

## Discussion

The marble burying test is often used as an indicator of anxiety and OCD (Broekkamp et al., 1986; Borsini et al., 2002; Thomas et al., 2009; Angoa-Pérez et al., 2013; Eltokhi et al., 2018). However, the meaning of marble burying behavior is highly debated throughout literature. Here, we introduce an analysis method that can increase inter-experimenter and intra-experimenter repeatability and establishes marble burying as its own unique behavior. We found that manual annotation of four 10-min-long videos by four independent observers blinded for genotype showed high variability in the identified average bout duration between observers, indicating that besides being time-consuming, manual annotation of burying bouts lacks reproducibility (Fig. 1*B*). We successfully trained a JAABA classifier by having skilled experimenters annotate video frames, which then independently scored burying behavior in *Ube3a^m-/p+^* mice consistent with existing literature (Fig. 2*A*).

A substantial benefit of automated classification is to allow for nonexperienced observers to score the marble burying results in a replicable manner that is consistent with skilled experimenters scoring the number of buried marbles (Fig. 7). Our classifier was able to score burying behavior in mice across varying laboratory settings, with differences in the bedding materials and behavioral boxes (wood chip bedding for *Ube3a* and *Shank2* groups, and corn cob bedding for *Sapap3* groups), which demonstrates its applicability across different behavioral setups. A classifier can be (re-)trained to accommodate varying experimental designs and conditions or to include additional objects. Together with spatial information from tracking data, this will allow for combining the study of marble burying behavior with paradigms such as novelty tests.

Additional information of mouse behavioral patterns gained from automated classification is an important step toward elucidating the biological meaning of marble burying. While a visual quantification of buried marbles at the end of each test results in a single output parameter (i.e., number of marbles buried), characterizing the actual behavior provides insight into specific burying characteristics and varying burying patterns over time. For example, *Sapap3*^−/−^ mice were previously shown to have a significant shift to longer grooming events and differentiating grooming probability over time (Ramírez-Armenta et al., 2022), however we did not find this tendency in the burying bouts characteristics during the marble burying test (Fig. 6*C*). In contrast, we found that the *Ube3a^m-/p+^* and *Shank2*^−/−^ mice showed a decrease in burying behavior over the entire duration of the test (Fig. 6*A*,*B*). This analysis is suitable for testing pharmacological, chemogenetic and optogenetic interventions, where tracking changes in the behavior over time is a crucial experimental output. Finally, the use of the classifier in combination with the tracking software reveals novel spatial-information parameters, previously unavailable in the marble burying test. Our analysis showed a striking decrease in *Ube3a^m-/p+^* specificity for burying in the corners of the arena (Fig. 2*D*). This was further seen after midazolam treatment. *Shank2*^−/−^ mice showed a decreased preference for the corners of the test after midazolam injection (Fig. 4*B*), consistent with its anxiolytic effects (Wise et al., 2012). Overall, the activity analysis shows that there is no correlation between the level of activity and burying behavior as *Shank2*^−/−^ mice show clear hyperactivity but less burying bouts compared with their WT littermates. This finding is in contrast to a recently published study (Berg et al., 2021), where the authors discuss low activity as a potential cause for decreased number of buried marbles in an AS mouse model. Our observations are however consistent with the recent study showing that rescue of the *Ube3a* expression in the juvenile mice alleviates the motor deficits in the rotarod assay but not the marble burying (Milazzo et al., 2021). Our pharmacological intervention with midazolam significantly reduced the hyperactivity in *Shank2*^−/−^ mice. However, this did not result in a rescue of the burying phenotype (Fig. 4*A*). Together, these data strongly indicate that motor activity is not directly related to the burying behavior in *Ube3a^m-/p+^* and *Shank2*^−/−^ mice.

For cases in which additional information about burying characteristics is not required, we introduced a custom-written ImageJ script for the analysis of buried marble surface area (Fig. 7*A*). This method showed consistent results with both the classifier (Fig. 7*B*) and visual scoring of buried marbles (Fig. 7*C*), and showed a strong correlation with visual scoring results (Fig. 7*E*, top). Burying behavior showed no clear correlation with the number of buried marbles on a per-animal basis (Fig. 7*E*, bottom). This disparity can be caused by factors such as marbles being buried and unburied several times over the duration of the test, which cannot be captured by the scoring of the resulting buried marbles. The lack of correlation between the burying events and the number of buried marbles further emphasizes the need for using the methods, such as our classifier, that directly measure the burying behavior instead of focusing solely on the outcome (buried marbles). Further research will need to confirm whether the disparity between buried marbles and burying bouts is present in mouse models showing increased burying behavior, or whether this pattern is specific for mice, which present with decreased burying behavior.

By combining the additional information gained from the classifier with selective mouse models known for repetitive and compulsive-like behaviors we aimed to test whether these phenotypes will cause deficits in the marble burying test. Previous studies have shown increased repetitive behavior and an obsessive-compulsive-like phenotype in *Shank2*^−/−^ and *Sapap3*^−/−^ mice, represented by the increased levels of self-grooming behavior (Welch et al., 2007; Schmeisser et al., 2012). In our experiments, *Shank2*^−/−^ mice showed a significant decrease in the number of burying bouts and overall burying time (Fig. 3*A*). No difference was found between *Sapap3*^−/−^ mice and their WT littermates (Fig. 4). These results indicate that repetitive behavior is not a unitary construct (Ehmer et al., 2020a) and that burying behavior, although it is often ascribed to repetitive behaviors (Thomas et al., 2009), captures a distinct behavioral aspect.

Assigning the marble burying behavior to its unique class, distinct from other behaviors, can explain not only our own results but many previously published confounding findings. Our current understanding of the behavioral implications of burying behavior are still far removed from being able to relate a phenotype to human conditions. Thus, more research is necessary to elucidate the exact biological background of burying behavior.

In conclusion, in this study, we provide a novel method for replicable analysis of the marble burying test by automated classification of behavior. The classifier scored decreased levels of *Ube3a^m-/p+^* burying behavior consistent with literature, providing a way to reduce inter-experimenter and intra-experimenter variability as well as allowing nonexperienced observers to accurately analyze the marble burying test. We provide a reproducible alternative in the form of an image analysis script for cases in which additional information is not required. Mouse models of ASD and OCD, which previously showed increased levels of repetitive behavior, were found to have decreased levels of burying behavior (*Shank2*^−/−^) or no difference from WTs (*Sapap3*^−/−^). Benzodiazepine treatment in *Shank2*^−/−^ mice showing hyperactivity reduced locomotor activity but did not rescue reduced burying behavior. Together, these data strongly indicate that motor activity is not directly related to the burying behavior.

## Synthesis

Reviewing Editor: Nathalie Ginovart, Universite de Geneve Decisions are customarily a result of the Reviewing Editor and the peer reviewers coming together and discussing their recommendations until a consensus is reached. When revisions are invited, a fact-based synthesis statement explaining their decision and outlining what is needed to prepare a revision will be listed below. The following reviewer(s) agreed to reveal their identity: Zoltán Sarnyai, De Wet Wolmarans.

Synthesis of review:

Upon discussion, the reviewers expressed interest in your manuscript and agreed on the significance of the work as a novel and valuable contribution to the field. However, they also raised some issues that should be addressed before publication. Importantly, there was a consensus that since no animal model perfectly replicates clinical conditions, the use of genetically modified mouse models of ASD and OCD may lack the face and construct validity for understanding the functional significance of marble burying activity. This aspect of the manuscript should therefore be tone-down and rephrased.

Please find the specific comments and queries raised by the reviewers below:

Reviewer 1 :

This manuscript addresses an important and timely issue, the objective, unbiased, and automated analysis of the marble burying behavior in mice. The authors are correct in identifying the problem both with the measurement and the interpretation (the behavioral “meaning”) of the marble burying. The results are convincing in identifying a machine learning application which is as good as, or better than, the trained human observer in identifying the marble burying and measuring the marble surface buried. These are already important advances.

Separating the potential impact of hyper- or hypo-activity from the marble burying behavior is another important new result. They identified that burying behavior/digging activity does not correlate with the number of marble buried and conclude that counting the number of marbles buried is probably a flawed approach on its own. It would be important to know if similar dissociation exists in mouse lines that were previously identified to have high marble burying activity through measuring the number of marbles buried. If it does, that would question all the past results on marble burying. Or, alternatively, it would question the new, AI-based algorithm presented in this manuscript.

In fact, one can raise that the marble burying behavior is not “intended” to bury the marbles. It is excessive digging in the presence of the marbles. We know that anxiety-like behavior-inducing stressful/painful stimuli, e.g. mild electric shock delivered by a rod protruding into the cage, elicits digging behavior. If marbles in the cage are stressful stimuli due to their novelty they can induce digging. Therefore, mice that habituate will decrease the burying activity over time as opposed to the mic that fail to habituate and show no decrease over time. These issues need to be discussed.

However, I find the use of the genetically modified mouse lines as validation of the functional “meaning” of the marble burying less convincing. The fact that their algorithm identifies high marble burying activity where that was already identified by previous studies using conventional approaches. This is a validation of the method itself. It shows that the lack of certain genes will result in, through thousands of unknown steps, an altered digging activity in the presence of marbles. That’s all we can honestly say here. Just because these mouse lines have been used to study ASD and OCD-like mechanisms we can’t quite conclude that we are measuring anything that is related to the human conditions. These models, as almost all the other animal models in psychiatry, fall short of one or more of the validation criteria, face, construct and predictive validity.

Using pharmacological approaches, although that would not have given the final answer either, would have been informative. For example, what are the effects of benzodiazepine anxiolytics (effective in generalized anxiety acutely) or SSRIs (somewhat effective in OCD)? If activity has nothing to do with the digging behavior (I am sure that is only up to a certain level of hypo- or hyper-activity) then antipsychotic effect would also be interesting and important (from ASDs point of view) to study.

Line 410-413: “These results indicate that compulsivity is not a unitary construct (Ehmer, Crown, et al., 2020) and that burying behavior, although it is often ascribed to repetitive behaviors (Thomas et al., 2009), captures a distinctly different behavioral aspect than self-grooming.” On what basis can we then determine “compulsivity”? Is it excessive self-grooming? Is it excessive digging behavior in the presence of marbles?

Do mice that show high marble burying activity (e.g. excessive digging in the presence of marbles) exhibit excessive digging in the home cage in the absence of the marbles or other novel objects? That would perhaps be a more appropriate test of “compulsivity”. Perhaps it is the difference between mice that bury excessively without the marbles and the once that require the marbles to be present for excessive burying behavior holds the key to sort out the “anxiety", “compulsivity” or “excessive stereotypic behavior” interpretations.

Reviewer 2 :

I commend the authors for a valuable body of work and for the generally detailed manner in which they reported their rationale and findings. The MS sets out to explore and deliver an automated, computer-assisted scoring method for the marble burying test, which is, as they state, highly debated in literature. However, there are some aspects of the MS that need further work.

In the abstract and throughout the arguing, it is stated that the MBT as is, solely rely on the number of marbles buried at the end of the test (see abstract, line 5 for example). This is not entirely true - the authors would do well do study detailed reviews on the topic that were published during the past few years. To simplify the dilemma which is the marble burying test in this manner, is not accurate and ignores the work of other laboratories that have also likely struggled with these questions in the past.

Introduction, lines 56-60 - This paragraph is oversimplified against the background of existing literature. The authors cite the work of Thomas et al, but ignores the multitude of other studies that showed the contrary. In fact, this is one of the problems of the test - it is applied with different methods and then cited out of context.

Introduction, lines 96 - 98 - The question of whether marble burying is purely compulsive-like or repetitive has been addressed in detail before, with several authors concluding that it is not the same. The authors would benefit from interrogating this literature before making this argument. I’d suggest the paragraph rather be rephrased along the lines of “... whether marble burying in these strains, would co-occur with other previously reported compulsive-like behaviors, e.g. excessive grooming.” Here and in the discussion, the authors provide detailed explanations of the differences between grooming and burying, but grooming was not investigated in this work. Thus, less emphasis should be placed on this.

Methods - Detail re the number of female and male animals used in each KO and WT cohort of each strain is needed.

Methods - Why were strains 1 and 2 kept on wood-chips and SAPAP mice on corncob? One of the major issues with the MBT is burying substrate. This reviewer is not convinced that woodchips is in any means suitable as such, while this has been argued and confirmed by other authors as well. Why were wood chips chosen? Also, based on prior in-depth analysis of the test, detailed descriptions of the burying substrates are needed - density, particle size and weight per 100g.

Methods - the average mouse weights of the different strains are needed. Is it possible that marbles spaced on a light bedding substrate like woodchips can be manipulated simply by moving, i.e. that burying bouts do not necessarily reflect marble-directed behavior?

Methods - one significant concern for me lies in the fact that while the classifier training was done by means of assisted supervision, the authors fail to address the inherent discrepancy between the scores of different observers. In other words, the fact that the classifier training can be subject to trainer/observer bias, itself, needs careful attention. Training would ultimately depend on the observer that trains the software, would it not?

Results, lines 217 - 218 - this statement needs reworking against the background of different observers generating different scores. Thus, the software correctly annotated certain behaviors according to what parameter?

Results, line 270 - This is an oversimplification. The results are and meaning of the marble burying test is clear, but depend on the type of experiment and how it is executed.

Results, line 298 and elsewhere - please refrain from referring to human behaviors in animals - keep with compulsive-like, persistent, repetitive etc.

Results - line 330 - when using woodchip bedding, surface area calculations as a potential measure of buried marbles, is redundant for logical reasons which have also been reviewed before. The authors need to address this, since their data confirm such (lines 335-336). I therefore disagree with lines 337 - 339 - there is no value in measuring this construct. Please remove.

Discussion, lines 360 - 363 - the issue of how and by whom training is provided to the classifier, needs attention.

Discussion, lines 374 - 377 - reference here and elsewhere to SAPAP KO mice need reworking, since it is posited, though not directly stated, that SAPAP KO mice must show some sort of combined grooming-burying behavior, which is refuted by using classifier scoring. Such a theory is not valid and I would simply suggest rephrasing the whole topic simply to say that the purpose of using the SAPAP KO’s, was to see whether they would present with another compulsive-like phenotype also.

Discussion, lines 410 - 418 - this paragraph is over-detailed and addresses a much deeper question. This should be removed.

## Author Response

Synthesis of Reviews:

Computational Neuroscience Model Code Accessibility Comments for Author (Required):

Links to open-source softwares are provided.

The URL to access the code described in the paper is given in the cover letter. The URL was checked and is working.

However, the statement under the “Code accessibility"subsection is incomplete in both the cover letter and in the MS as the mention “The code is available as Extended Data” is lacking.

Moreover, as far as I can see, no extended data have been provided with the submission.

To comply with the Policy on Computational neuroscience computer code, the authors should therefore: 1) complete in full the statement regarding code accessibility in the Materials and Methods section, and 2) upload the code as Extended Data to the MS.

We have edited the “Code accessibility” subsection of the cover letter and manuscript to include the full statement and have added the code to the extended data. The classifier cannot be uploaded as a text file code and therefore was deposited at G-Node GIN.

Significance Statement Comments for Author (Required):

N/A

Comments on the Visual Abstract for Author (Required):

Synthesis Statement for Author (Required):

Synthesis of review:

Upon discussion, the reviewers expressed interest in your manuscript and agreed on the significance of the work as a novel and valuable contribution to the field. However, they also raised some issues that should be addressed before publication. Importantly, there was a consensus that since no animal model perfectly replicates clinical conditions, the use of genetically modified mouse models of ASD and OCD may lack the face and construct validity for understanding the functional significance of marble burying activity. This aspect of the manuscript should therefore be tone-down and rephrased.

Please find the specific comments and queries raised by the reviewers below:

We thank the reviewers for the careful attention they gave to our manuscript and constructive feedback. The reviewers’ suggested revisions are a substantial improvement on our manuscript and we feel that they have significantly strengthened our results and conclusions. We have edited the text throughout the manuscript to reflect reviewers’ comments and have added a suggested neuropharmacological experiment using benzodiazepines in the Result section.

Our point­-by-­point response to comments is given below.

This manuscript addresses an important and timely issue, the objective, unbiased, and automated analysis of the marble burying behavior in mice. The authors are correct in identifying the problem both with the measurement and the interpretation (the behavioral “meaning”) of the marble burying. The results are convincing in identifying a machine learning application which is as good as, or better than, the trained human observer in identifying the marble burying and measuring the marble surface buried. These are already important advances.

We thank the reviewers for the recognition of the importance of our work.

Separating the potential impact of hyper- or hypo-activity from the marble burying behavior is another important new result. They identified that burying behavior/digging activity does not correlate with the number of marble buried and conclude that counting the number of marbles buried is probably a flawed approach on its own. It would be important to know if similar dissociation exists in mouse lines that were previously identified to have high marble burying activity through measuring the number of marbles buried. If it does, that would question all the past results on marble burying. Or, alternatively, it would question the new, AI-based algorithm presented in this manuscript.

This is indeed of interest, by showing that this novel way of analysis can be used on mice with varying phenotypes in the current methods paper, we hope that these questions can be adequately studied. We added this point to the discussion.

In fact, one can raise that the marble burying behavior is not “intended” to bury the marbles. It is excessive digging in the presence of the marbles. We know that anxiety-like behavior-inducing stressful/painful stimuli, e.g. mild electric shock delivered by a rod protruding into the cage, elicits digging behavior. If marbles in the cage are stressful stimuli due to their novelty they can induce digging. Therefore, mice that habituate will decrease the burying activity over time as opposed to the mice that fail to habituate and show no decrease over time. These issues need to be discussed.

It has been shown previously in Taylor et al., 2017 and Thomas et al., 2009 that mice do not decrease burying activity with repeated testing over multiple days, but do decrease burying when tested 5x within 1 day. This can indicate that burying is not considered an anxiety-like phenotype elicited by the marbles. If the behavior is indeed not intended to bury the marbles, then this is in support of our switch to an automated classification system where we study the behavior itself and not the marbles. We have added this discussion to the introduction.

However, I find the use of the genetically modified mouse lines as validation of the functional “meaning” of the marble burying less convincing. The fact that their algorithm identifies high marble burying activity where that was already identified by previous studies using conventional approaches. This is a validation of the method itself. It shows that the lack of certain genes will result in, through thousands of unknown steps, an altered digging activity in the presence of marbles. That’s all we can honestly say here. Just because these mouse lines have been used to study ASD and OCD-like mechanisms we can’t quite conclude that we are measuring anything that is related to the human conditions. These models, as almost all the other animal models in psychiatry, fall short of one or more of the validation criteria, face, construct and predictive validity.

This is an important and subtle point. We agree that our current understanding is not enough to relate any functional ’meaning’ of marble burying behavior to neurodevelopmental disorders such as ASD or OCD. Hence, we have made sure to include no statements linking burying behavior to ASD or OCD in humans. Further we specifically chose the Shank2 and AS models as they have been previously studied in the MB test and are used in several preclinical studies.

However, the question regarding the usefulness of mouse models for human neurodevelopmental and psychiatric conditions is a part of a bigger discussion in the neuroscience field. In fact, many prominent scientist such as Huda Zoghbi (https://pubmed.ncbi.nlm.nih.gov/30030131/), Jacqueline Crawley (https://www.ncbi.nlm.nih.gov/pmc/articles/PMC3513683/) or Mustafa Sahin (https://www.sciencedirect.com/science/article/pii/S096098221830976X) see a promise in combining mouse models with other translational tools and “will continue to drive disease gene discovery and lead to better understanding of gene functions and disease mechanisms, laying the groundwork for future therapeutic interventions.” We added a clarifying sentence in the discussion regarding this point.

Using pharmacological approaches, although that would not have given the final answer either, would have been informative. For example, what are the effects of benzodiazepine anxiolytics (effective in generalized anxiety acutely) or SSRIs (somewhat effective in OCD)? If activity has nothing to do with the digging behavior (I am sure that is only up to a certain level of hypo- or hyper-activity) then antipsychotic effects would also be interesting and important (from ASDs point of view) to study.

We agree with the reviewer. We have now added experiments to test the effects of benzodiazepine anxiolytic injections on the hyperactivity and burying phenotype (New Figure 4).

To account for the possible changes in the behavior after benzodiazepines administration we additionally trained the classifier on a mouse data obtained from this experiment. This did not change the accuracy of the classifier but ensured that it is robust and correctly classifies longer periods of inactivity. The frame and mouse numbers are updated in the manuscript.

Line 410-413: “These results indicate that compulsivity is not a unitary construct (Ehmer, Crown, et al., 2020) and that burying behavior, although it is often ascribed to repetitive behaviors (Thomas et al., 2009), captures a distinctly different behavioral aspect than self-grooming.” On what basis can we then determine “compulsivity”? Is it excessive self-grooming? Is it excessive digging behavior in the presence of marbles?

We have replaced “compulsivity” with “repetitive behavior", as the current study does not provide a complete assessment of what a “compulsivity” phenotype in mice entails.

Do mice that show high marble burying activity (e.g. excessive digging in the presence of marbles) exhibit excessive digging in the home cage in the absence of the marbles or other novel objects? That would perhaps be a more appropriate test of “compulsivity”. Perhaps it is the difference between mice that bury excessively without the marbles and the once that require the marbles to be present for excessive burying behavior holds the key to sort out the “anxiety", “compulsivity” or “excessive stereotypic behavior” interpretations.

This is an intriguing possibility, which can be studied by training a classifier on a configuration that includes other novel objects. We have added this to the Discussion.

Reviewer 2 :

I commend the authors for a valuable body of work and for the generally detailed manner in which they reported their rationale and findings. The MS sets out to explore and deliver an automated, computer-assisted scoring method for the marble burying test, which is, as they state, highly debated in literature. However, there are some aspects of the MS that need further work.

In the abstract and throughout the arguing, it is stated that the MBT as is, solely rely on the number of marbles buried at the end of the test (see abstract, line 5 for example). This is not entirely true - the authors would do well do study detailed reviews on the topic that were published during the past few years. To simplify the dilemma which is the marble burying test in this manner, is not accurate and ignores the work of other laboratories that have also likely struggled with these questions in the past.

Although several previous studies have struggled with the analysis of the marble burying test, this resulted in the addition of analyzing the inter-observer reliability (Kinsey et. al, 2011) or manual post-experimental assessment of photographs (Homma et. al, 2009). Both methods however still rely on manual scoring of the marbles and not the behavior. The analysis of the marble burying test is widely considered to rely solely on manual scoring, which has been stated in several (recent) reviews discussing the marble burying test; “Because scoring in the MBT is always performed manually (...)” (de Brouwer et. al, 2019). We have added these citations to the introduction.

Introduction, lines 56-60 - This paragraph is oversimplified against the background of existing literature. The authors cite the work of Thomas et al, but ignores the multitude of other studies that showed the contrary. In fact, this is one of the problems of the test - it is applied with different methods and then cited out of context.

As stated in the previous comment, we have added citations to other papers.

Introduction, lines 96 - 98 - The question of whether marble burying is purely compulsive-like or repetitive has been addressed in detail before, with several authors concluding that it is not the same. The authors would benefit from interrogating this literature before making this argument. I’d suggest the paragraph rather be rephrased along the lines of “... whether marble burying in these strains, would co-occur with other previously reported compulsive-like behaviors, e.g. excessive grooming.” Here and in the discussion, the authors provide detailed explanations of the differences between grooming and burying, but grooming was not investigated in this work. Thus, less emphasis should be placed on this.

We agree with the concerns raised by the reviewer. We have revised the introduction and discussion to focus on burying behavior versus movement characteristics.

Methods - Detail re the number of female and male animals used in each KO and WT cohort of each strain is needed.

We apologize for this omission. We have now added information regarding the number of female and male mice in methods.

Methods - Why were strains 1 and 2 kept on wood-chips and SAPAP mice on corncob? One of the major issues with the MBT is burying substrate. This reviewer is not convinced that woodchips is in any means suitable as such, while this has been argued and confirmed by other authors as well. Why were wood chips chosen? Also, based on prior in-depth analysis of the test, detailed descriptions of the burying substrates are needed - density, particle size and weight per 100g.

Woodchips were chosen as this is the substrate which has been used previously in experiments which tested Ube3a mutant mice. As this is the “validation” method for the current study, experimental factors were kept consistent with previous work for an accurate comparison. Density of woodchip bedding was 204g/L, which has now been added to the text. There are many commercially available bedding materials. Yamada et. al, 2009, figure 1 shows density measurements for several bedding materials used in the marble burying test, where our woodchip bedding would fall on the denser side of the spectrum of materials.

Sapap3 mutant mice and their wildtype littermates however were housed on corncob bedding in their respective institute and thus tested on this material. This bedding material had a density of 566 g/L.

Methods - the average mouse weights of the different strains are needed. Is it possible that marbles spaced on a light bedding substrate like woodchips can be manipulated simply by moving, i.e. that burying bouts do not necessarily reflect marble-directed behavior?

Weight is a feature that is unfortunately commonly omitted in discussion of marble burying results. Therefore, moving to an analysis of quantifying burying behavior as shown in this manuscript is preferable from analyzing the marbles as a proxy, as the analysis of burying bouts is independent of mouse weights. It is an interesting point and especially important in for example obesity studies where larger differences in weights are expected. We added the average weight of different strains to the Methods

Methods - one significant concern for me lies in the fact that while the classifier training was done by means of assisted supervision, the authors fail to address the inherent discrepancy between the scores of different observers. In other words, the fact that the classifier training can be subject to trainer/observer bias, itself, needs careful attention. Training would ultimately depend on the observer that trains the software, would it not?

Indeed, anything trained or scored by observers can be affected by observer bias. The important difference with automated classification, is that experiments can be scored consistently over time, even when experimenters change, by using the same classifier. This method is particularly helpful when there is a high number of employees/ students leaving and joining a laboratory. This consistency is especially valuable in analyzing additional experiments for revisions consistent with previous data. Additionally, wildtype and mutant mice can be scored without bias due to previous knowledge regarding the genotype, as in some cases it is very obvious to the observer (different coat color, hyperactivity or altered gait make it impossible to blind the genotype for the researchers).

Results, lines 217 - 218 - this statement needs reworking against the background of different observers generating different scores. Thus, the software correctly annotated certain behaviors according to what parameter?

The accuracy is obtained by cross-validation, where labels are removed for a subset of frames used in training the classifier. The software then uses the classifier to make predictions for these frames which then are compared to the original labels given by the trained observers during training of the classifier. We have added a description of this process to the methods.

Results, line 270 - This is an oversimplification. The results are and meaning of the marble burying test is clear, but depend on the type of experiment and how it is executed.

As stated in de Brouwer et. al, 2019, we have clarified by rewording to emphasize that the consensus regarding the interpretation is lacking.

Results, line 298 and elsewhere - please refrain from referring to human behaviors in animals - keep with compulsive-like, persistent, repetitive etc.

We have reworded the statements in the results and elsewhere to refrain from referring to human behaviors in animals.

Results - line 330 - when using woodchip bedding, surface area calculations as a potential measure of buried marbles, is redundant for logical reasons which have also been reviewed before. The authors need to address this, since their data confirm such (lines 335-336). I therefore disagree with lines 337 - 339 - there is no value in measuring this construct. Please remove.

The current method of analyzing the marble burying test relies solely on visual scoring. We agree that since measuring surface area does not fully correlate with burying behavior, this would not be our preferred method for analysis. However, measuring surface area showed a strong correlation with the current analysis method (visual scoring, Figure 6E, top), with the added benefit of standardization and repeatability, making this a preferred method over solely visual scoring using the current method.

We introduce both methods that can measure both the burying behavior and the output. Other users can choose the preferred scoring method.

Discussion, lines 360 - 363 - the issue of how and by whom training is provided to the classifier, needs attention.

We have added a clarification to the lines mentioned by the reviewer stating that the classifier was initially trained by skilled experimenters, which can henceforth be used by non-skilled observers to score burying in a repeatable way.

Discussion, lines 374 - 377 - reference here and elsewhere to SAPAP KO mice need reworking, since it is posited, though not directly stated, that SAPAP KO mice must show some sort of combined grooming-burying behavior, which is refuted by using classifier scoring. Such a theory is not valid and I would simply suggest rephrasing the whole topic simply to say that the purpose of using the SAPAP KO’s, was to see whether they would present with another compulsive-like phenotype also.

-We agree with the concerns raised by the reviewer. We have revised this section of the discussion.

Discussion, lines 410 - 418 - this paragraph is over-detailed and addresses a much deeper question. This should be removed.

-We agree with the comment of the reviewer. We have removed the section concerning the interpretation of grooming behavior and focused on burying behavior.
